# Crystal structure of di-μ-iodido-bis­{[1,3-bis­(2,6-diiso­propyl­phen­yl)imidazol-2-yl­idene]lithium}

**DOI:** 10.1107/S2056989015009822

**Published:** 2015-05-28

**Authors:** Hui-Da Wan, Jian-Quan Hong

**Affiliations:** a School of Chemical and Material Engineering, Jiangnan University, 1800 Lihu Road, Wuxi, Jiangsu Province 214122, People’s Republic of China

**Keywords:** crystal structure, dinuclear lithium complex, imidazol-2-yl­idene ligand, catalysis

## Abstract

In the title binuclear complex, [Li_2_(C_27_H_36_N_2_)_2_I_2_], the unique Li^I^ cation is coordinated by two iodide anions and one yl­idene C atom from a 1,3-bis­(2,6-diiso­propyl­phen­yl)imidazol-2-yl­idene ligand in a distorted trigonal–planar geometry. The two symmetry-related iodide anions bridge two Li^I^ cations, forming an inversion dimer in which the Li_2_I_2_ plane is nearly perpendicular to the imidazol-2-yl­idene ring, with a dihedral angle of 85.5 (3)°. No hydrogen bonding is observed in the crystal.

## Related literature   

For a related lithium complex of imidazol-2-ylidenes, see: Hill *et al.* (2011 ▸). For related lithium complexes with Li—I bonds, see: Raston *et al.* (1989 ▸); Fei *et al.* (2003 ▸); Thatcher *et al.* (2012 ▸). For applications of imidazol-2-ylidenes in catalysis, see: Vougioukalakis & Grubbs (2010 ▸); Fortman & Nolan (2011 ▸); Valente *et al.* (2012 ▸); Riener *et al.* (2014 ▸); Wang *et al.* (2008 ▸); Mahoney *et al.* (2013 ▸); Kolychev *et al.* (2013 ▸); Biju *et al.* (2011 ▸); Berkessel *et al.* (2012 ▸); Fèvre *et al.* (2013 ▸).
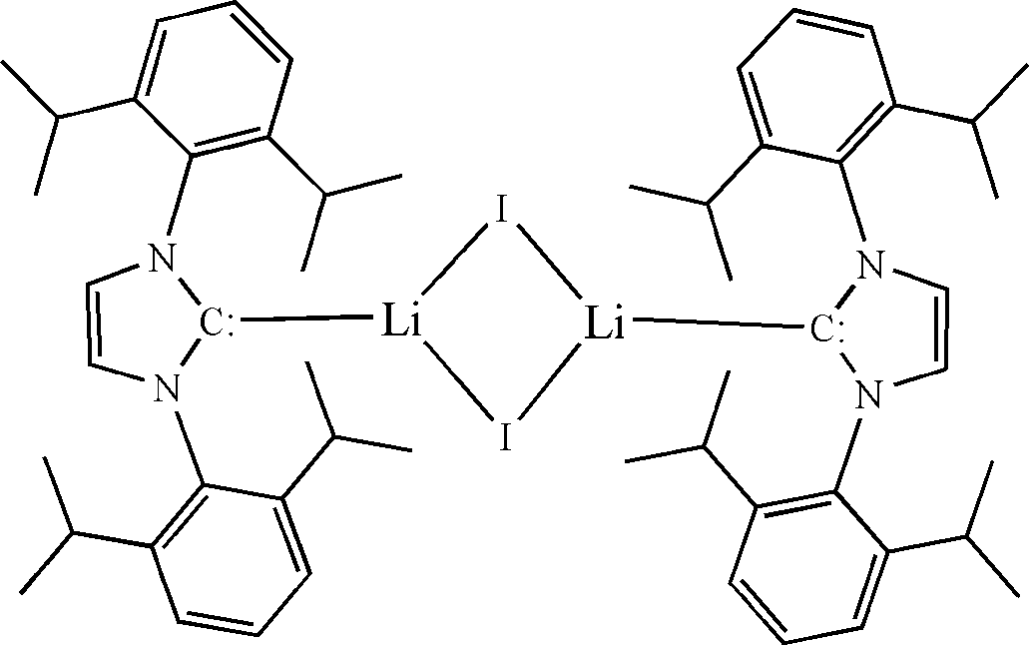


## Experimental   

### Crystal data  

[Li_2_(C_27_H_36_N_2_)_2_I_2_]
*M* *_r_* = 1044.84
Monoclinic, 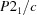

*a* = 10.645 (4) Å
*b* = 14.490 (6) Å
*c* = 19.217 (7) Å
β = 105.565 (6)°
*V* = 2855.4 (19) Å^3^
*Z* = 2
Mo *K*α radiation
μ = 1.14 mm^−1^
*T* = 293 K
0.30 × 0.25 × 0.20 mm

### Data collection  

Bruker APEXII CCD diffractometer
Absorption correction: multi-scan (*SADABS*; Bruker, 2007 ▸) *T*_min_ = 0.727, *T*_max_ = 0.805
11711 measured reflections
5069 independent reflections
2924 reflections with *I* > 2σ(*I*)
*R*_int_ = 0.085

### Refinement  

*R*[*F* ^2^ > 2σ(*F* ^2^)] = 0.054
*wR*(*F* ^2^) = 0.112
*S* = 1.00
5069 reflections
288 parameters
H-atom parameters constrained
Δρ_max_ = 0.74 e Å^−3^
Δρ_min_ = −1.23 e Å^−3^

### 

Data collection: *APEX2* (Bruker, 2007 ▸); cell refinement: *SAINT* (Bruker, 2007 ▸); data reduction: *SAINT*; program(s) used to solve structure: *SHELXS97* (Sheldrick, 2008 ▸); program(s) used to refine structure: *SHELXL97* (Sheldrick, 2008 ▸); molecular graphics: *SHELXTL* (Sheldrick, 2008 ▸); software used to prepare material for publication: *SHELXTL*.

## Supplementary Material

Crystal structure: contains datablock(s) I, New_Global_Publ_Block. DOI: 10.1107/S2056989015009822/xu5851sup1.cif

Structure factors: contains datablock(s) I. DOI: 10.1107/S2056989015009822/xu5851Isup2.hkl

**Fig. 1.**
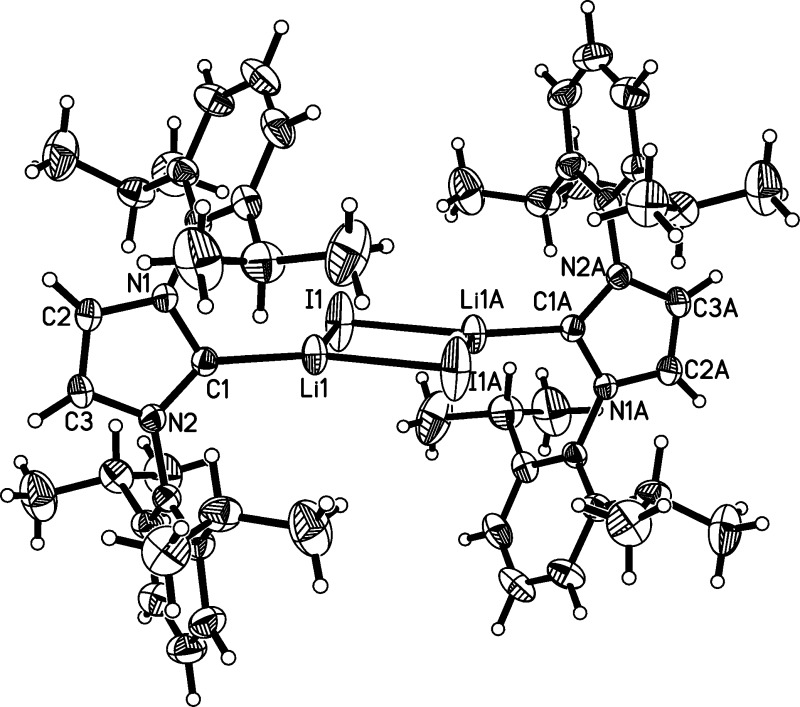
The molecular structure of the title compound with the atom-numbering scheme and 30% probability displacement ellipsoids.

**Fig. 2.**
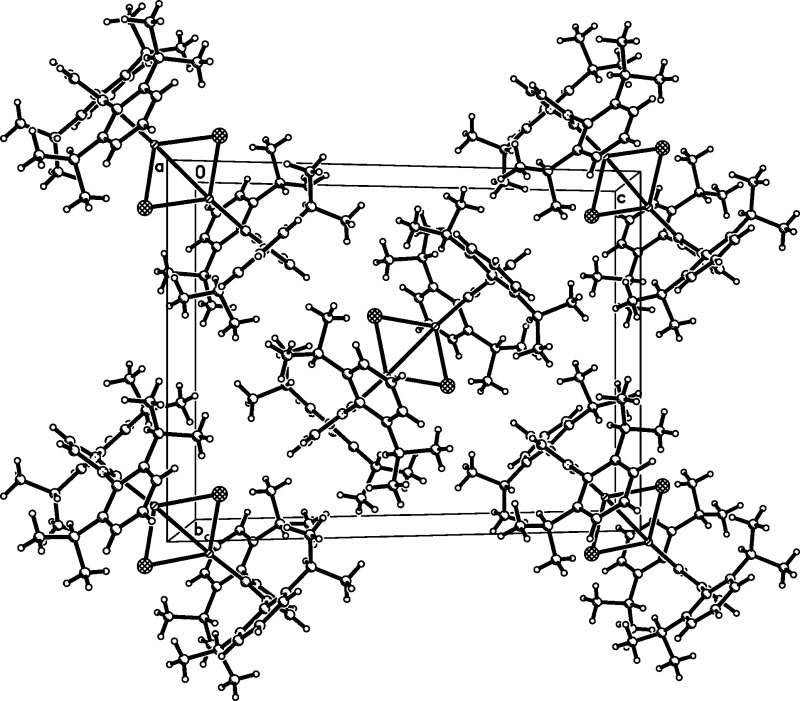
The packing diagram viewed along the a axis.

. DOI: 10.1107/S2056989015009822/xu5851fig1.tif
The mol­ecular structure of the title compound with the atom-numbering scheme and 30% probability displacement ellipsoids.

a . DOI: 10.1107/S2056989015009822/xu5851fig2.tif
The packing diagram viewed along the *a* axis.

CCDC reference: 1402139

Additional supporting information:  crystallographic information; 3D view; checkCIF report

## Figures and Tables

**Table 1:** Selected bond lengths ()

Li1C1	2.120(7)
Li1I1	2.676(8)
Li1I1^i^	2.691(7)

Symmetry code: (i) 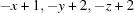.

## supplementary crystallographic information

### S1. Comment

Imidazol-2-ylidenes (NHCs), are excellent σ-donors and readily coordinate to transition metals and p-block elements. This feature has led to the most important application of NHCs as ancillary ligands in homogeneous transition-metal catalysis (Vougioukalakis *et al.*, 2010; Fortman *et al.*, 2011; Valente *et al.*, 2012; Riener *et al.*, 2014) and *p*-block elements (Wang *et al.*, 2008; Mahoney *et al.*, 2013; Kolychev *et al.*, 2013). NHCs have enabled the preparation and characterization of previously unknown species featuring p-block species in unconventional forms, such as in the zero-oxidation state or as radicals. As organocatalysts, NHCs can also promote a wide range of different organic transformations, with most processes involving an initial attack of the NHC onto a carbonyl group (Biju *et al.*, 2011; Berkessel *et al.*, 2012; Fèvre *et al.*, 2013).

Single-crystal X-ray diffraction analyses the title compound reveals that Li is tri-coordinate. As shown in Fig. 1, the moiety of the dimer contains one Li atom, a NHC ligand and one iodine atom. The dative Li← C bond distance is 2.120 (7) Å, whic is slightly shorter than those of NHC species [M(IPr)_2_]^+^[M'{N(SiMe_3_)_2_}_3_]^-^ (M = Li, Na, K; M' = Mg, Ca, Sr, Ba). (Hill *et al.*, 2011) The Li—I bond distances are in the range of 2.676 (8)–2.691 (7) Å, which are remarkably shorter than the values in lithium complexes (2.767 (16)–2.932 (6) Å) with µ-bridging iodine constructions. (Raston *et al.*, 1989; Fei *et al.*, 2003; Thatcher *et al.*, 2012). Fig. 2 shows the molecular packing of the title compound, viewed along the *a* axis.

### S2. Experimental

A flame-dried Schlenk tube under a nitrogen atmosphere was charged with 1,3-Bis(2,6-diisopropylphenyl)imidazolinium iodide (0.516 g, 1mmol) and THF (10mL) at -78°C. To this was added a THF solution of lithium hexamethyldisilazide (0.167 g, 1 mmol). The mixture was allowed to slowly warm to room temperature and was stirred for 3 h. All volatiles were removed under vacuum. The resulting pale-yellow material was washed with cold hexane quickly, and then dried up. 0.376 g (72 %) of [Li(I)(C_27_H_36_N_2_)]_2_ was obtained. Crystals suitable for X-ray analysis were obtained by recrystallization in THF/hexane at -30 °C. Elemental analysis (%) calcd. for Li_2_C_54_H_72_N_4_I_2_: C 62.07, H 6.94, N 5.36. Found: C 62.45, H 6.80, N 5.48.

### S3. Refinement

The H atoms bonded to C atoms were introduced at calculated positions and refined using a riding model, with U_iso_(H) = 1.2U_eq_(C) and C–H distances of 0.93–0.96 Å.

### Crystal data

**Table:**

[Li_2_(C_27_H_36_N_2_)_2_I_2_]	*F*(000) = 1072
*M**_r_* = 1044.84	*D*_x_ = 1.215 Mg m^−^^3^
Monoclinic, *P*2_1_/*c*	Mo *K*α radiation, λ = 0.71073 Å
Hall symbol: -P 2ybc	Cell parameters from 3834 reflections
*a* = 10.645 (4) Å	θ = 2.4–22.6°
*b* = 14.490 (6) Å	µ = 1.14 mm^−^^1^
*c* = 19.217 (7) Å	*T* = 293 K
β = 105.565 (6)°	Block, pale-yellow
*V* = 2855.4 (19) Å^3^	0.30 × 0.25 × 0.20 mm
*Z* = 2	

### Data collection

**Table:**

Bruker APEXII CCD diffractometer	5069 independent reflections
Radiation source: fine-focus sealed tube	2924 reflections with *I* > 2σ(*I*)
Graphite monochromator	*R*_int_ = 0.085
φ and ω scans	θ_max_ = 25.1°, θ_min_ = 1.8°
Absorption correction: multi-scan (*SADABS*; Bruker, 2007)	*h* = −12→12
*T*_min_ = 0.727, *T*_max_ = 0.805	*k* = −16→17
11711 measured reflections	*l* = −22→14

### Refinement

**Table:**

Refinement on *F*^2^	Primary atom site location: structure-invariant direct methods
Least-squares matrix: full	Secondary atom site location: difference Fourier map
*R*[*F*^2^ > 2σ(*F*^2^)] = 0.054	Hydrogen site location: inferred from neighbouring sites
*wR*(*F*^2^) = 0.112	H-atom parameters constrained
*S* = 1.00	*w* = 1/[σ^2^(*F*_o_^2^) + (0.0129*P*)^2^ + 2.6*P*] where *P* = (*F*_o_^2^ + 2*F*_c_^2^)/3
5069 reflections	(Δ/σ)_max_ = 0.002
288 parameters	Δρ_max_ = 0.74 e Å^−^^3^
0 restraints	Δρ_min_ = −1.23 e Å^−^^3^

### Special details

**Table:**

Geometry. All e.s.d.'s (except the e.s.d. in the dihedral angle between two l.s. planes) are estimated using the full covariance matrix. The cell e.s.d.'s are taken into account individually in the estimation of e.s.d.'s in distances, angles and torsion angles; correlations between e.s.d.'s in cell parameters are only used when they are defined by crystal symmetry. An approximate (isotropic) treatment of cell e.s.d.'s is used for estimating e.s.d.'s involving l.s. planes.
Refinement. Refinement of *F*^2^ against ALL reflections. The weighted *R*-factor *wR* and goodness of fit *S* are based on *F*^2^, conventional *R*-factors *R* are based on *F*, with *F* set to zero for negative *F*^2^. The threshold expression of *F*^2^ > σ(*F*^2^) is used only for calculating *R*-factors(gt) *etc*. and is not relevant to the choice of reflections for refinement. *R*-factors based on *F*^2^ are statistically about twice as large as those based on *F*, and *R*- factors based on ALL data will be even larger.

### Fractional atomic coordinates and isotropic or equivalent isotropic displacement parameters (Å^2^)

**Table:**

	*x*	*y*	*z*	*U*_iso_*/*U*_eq_	
Li1	0.5511 (8)	0.9291 (5)	0.9451 (4)	0.073 (2)	
N1	0.5147 (3)	0.7930 (2)	0.81899 (15)	0.0471 (8)	
N2	0.7164 (3)	0.8253 (2)	0.85073 (15)	0.0495 (8)	
I1	0.45770 (6)	1.10068 (3)	0.918028 (19)	0.1292 (3)	
C1	0.6043 (4)	0.8421 (3)	0.86912 (18)	0.0465 (9)	
C2	0.5699 (4)	0.7470 (3)	0.7712 (2)	0.0613 (11)	
H2	0.5272	0.7092	0.7331	0.074*	
C3	0.6961 (5)	0.7677 (3)	0.7908 (2)	0.0614 (12)	
H3	0.7589	0.7475	0.7687	0.074*	
C4	0.3808 (4)	0.7834 (3)	0.81913 (19)	0.0490 (10)	
C5	0.3503 (5)	0.7218 (3)	0.8690 (2)	0.0620 (11)	
C6	0.2220 (5)	0.7133 (4)	0.8677 (3)	0.0815 (15)	
H6	0.1990	0.6744	0.9008	0.098*	
C7	0.1267 (5)	0.7603 (4)	0.8191 (3)	0.0898 (17)	
H7	0.0398	0.7519	0.8185	0.108*	
C8	0.1584 (5)	0.8200 (3)	0.7711 (3)	0.0780 (14)	
H8	0.0924	0.8520	0.7385	0.094*	
C9	0.2867 (4)	0.8334 (3)	0.7702 (2)	0.0587 (11)	
C10	0.4551 (5)	0.6667 (4)	0.9205 (3)	0.0937 (17)	
H10	0.5353	0.7035	0.9313	0.112*	
C11	0.4827 (7)	0.5777 (5)	0.8879 (4)	0.148 (3)	
H11A	0.5586	0.5493	0.9192	0.222*	
H11B	0.4980	0.5896	0.8417	0.222*	
H11C	0.4093	0.5371	0.8820	0.222*	
C12	0.4245 (8)	0.6461 (5)	0.9924 (3)	0.153 (3)	
H12A	0.5024	0.6257	1.0271	0.229*	
H12B	0.3594	0.5987	0.9854	0.229*	
H12C	0.3927	0.7010	1.0098	0.229*	
C13	0.3189 (5)	0.9005 (3)	0.7174 (2)	0.0703 (13)	
H13	0.4135	0.9100	0.7314	0.084*	
C14	0.2809 (7)	0.8614 (5)	0.6418 (3)	0.127 (2)	
H14A	0.2993	0.9059	0.6089	0.190*	
H14B	0.1894	0.8474	0.6282	0.190*	
H14C	0.3298	0.8061	0.6404	0.190*	
C15	0.2544 (6)	0.9935 (4)	0.7182 (3)	0.112 (2)	
H15A	0.2695	1.0144	0.7671	0.168*	
H15B	0.1623	0.9878	0.6966	0.168*	
H15C	0.2904	1.0372	0.6912	0.168*	
C16	0.8436 (4)	0.8581 (3)	0.8895 (2)	0.0534 (10)	
C17	0.8907 (4)	0.9376 (3)	0.8648 (2)	0.0611 (11)	
C18	1.0158 (5)	0.9638 (3)	0.9004 (3)	0.0814 (14)	
H18	1.0511	1.0160	0.8847	0.098*	
C19	1.0898 (5)	0.9153 (4)	0.9582 (3)	0.0869 (16)	
H19	1.1738	0.9348	0.9816	0.104*	
C20	1.0385 (5)	0.8372 (4)	0.9814 (3)	0.0823 (15)	
H20	1.0892	0.8043	1.0205	0.099*	
C21	0.9143 (4)	0.8066 (3)	0.9482 (2)	0.0645 (12)	
C22	0.8123 (5)	0.9924 (3)	0.8010 (2)	0.0720 (13)	
H22	0.7264	0.9631	0.7854	0.086*	
C23	0.8713 (6)	0.9880 (5)	0.7376 (3)	0.135 (3)	
H23A	0.8821	0.9247	0.7258	0.202*	
H23B	0.9546	1.0182	0.7502	0.202*	
H23C	0.8144	1.0183	0.6967	0.202*	
C24	0.7897 (6)	1.0907 (3)	0.8207 (3)	0.1030 (18)	
H24A	0.8717	1.1226	0.8353	0.155*	
H24B	0.7493	1.0909	0.8598	0.155*	
H24C	0.7338	1.1212	0.7796	0.155*	
C25	0.8619 (5)	0.7196 (4)	0.9735 (3)	0.0839 (15)	
H25	0.7715	0.7119	0.9446	0.101*	
C26	0.9377 (6)	0.6344 (4)	0.9617 (3)	0.112 (2)	
H26A	1.0273	0.6404	0.9888	0.168*	
H26B	0.9329	0.6286	0.9113	0.168*	
H26C	0.9008	0.5804	0.9775	0.168*	
C27	0.8602 (6)	0.7278 (5)	1.0525 (3)	0.134 (3)	
H27A	0.8199	0.6740	1.0662	0.200*	
H27B	0.8117	0.7817	1.0585	0.200*	
H27C	0.9480	0.7329	1.0825	0.200*	

### Atomic displacement parameters (Å^2^)

**Table:**

	*U*^11^	*U*^22^	*U*^33^	*U*^12^	*U*^13^	*U*^23^
Li1	0.100 (6)	0.067 (5)	0.061 (4)	0.008 (5)	0.038 (4)	−0.015 (4)
N1	0.048 (2)	0.0510 (19)	0.0438 (17)	0.0020 (16)	0.0147 (16)	−0.0048 (15)
N2	0.049 (2)	0.053 (2)	0.0468 (17)	0.0063 (17)	0.0124 (16)	−0.0039 (15)
I1	0.2440 (6)	0.0875 (3)	0.0756 (2)	0.0609 (3)	0.0765 (3)	0.0164 (2)
C1	0.050 (3)	0.047 (2)	0.043 (2)	0.008 (2)	0.0126 (19)	0.0010 (17)
C2	0.064 (3)	0.067 (3)	0.055 (2)	−0.001 (2)	0.021 (2)	−0.019 (2)
C3	0.063 (3)	0.068 (3)	0.059 (3)	0.006 (2)	0.027 (2)	−0.017 (2)
C4	0.047 (3)	0.051 (2)	0.052 (2)	0.000 (2)	0.020 (2)	−0.0083 (18)
C5	0.069 (3)	0.060 (3)	0.064 (3)	0.000 (2)	0.030 (2)	−0.002 (2)
C6	0.090 (4)	0.077 (4)	0.096 (4)	−0.014 (3)	0.057 (3)	−0.003 (3)
C7	0.071 (4)	0.081 (4)	0.137 (5)	−0.004 (3)	0.063 (4)	−0.012 (4)
C8	0.056 (3)	0.071 (3)	0.112 (4)	0.012 (3)	0.030 (3)	0.009 (3)
C9	0.051 (3)	0.056 (3)	0.070 (3)	0.003 (2)	0.018 (2)	−0.006 (2)
C10	0.098 (4)	0.098 (4)	0.087 (3)	−0.014 (4)	0.028 (3)	0.038 (3)
C11	0.175 (7)	0.142 (7)	0.142 (6)	0.093 (6)	0.067 (5)	0.052 (5)
C12	0.254 (9)	0.122 (5)	0.081 (4)	−0.016 (6)	0.044 (5)	0.028 (4)
C13	0.058 (3)	0.069 (3)	0.081 (3)	0.006 (2)	0.012 (2)	0.019 (2)
C14	0.186 (7)	0.124 (5)	0.081 (4)	−0.022 (5)	0.055 (4)	0.001 (4)
C15	0.134 (5)	0.077 (4)	0.128 (5)	0.016 (4)	0.040 (4)	0.030 (4)
C16	0.046 (3)	0.059 (3)	0.054 (2)	0.008 (2)	0.011 (2)	−0.009 (2)
C17	0.053 (3)	0.054 (3)	0.075 (3)	0.005 (2)	0.014 (2)	−0.008 (2)
C18	0.073 (4)	0.064 (3)	0.105 (4)	−0.004 (3)	0.021 (3)	−0.009 (3)
C19	0.059 (3)	0.087 (4)	0.105 (4)	−0.004 (3)	0.005 (3)	−0.027 (3)
C20	0.066 (4)	0.099 (4)	0.069 (3)	0.015 (3)	−0.004 (3)	−0.008 (3)
C21	0.057 (3)	0.070 (3)	0.062 (3)	0.009 (3)	0.007 (2)	−0.004 (2)
C22	0.076 (3)	0.058 (3)	0.084 (3)	0.011 (3)	0.025 (3)	0.009 (2)
C23	0.160 (6)	0.153 (6)	0.105 (4)	0.076 (5)	0.060 (4)	0.036 (4)
C24	0.128 (5)	0.072 (4)	0.111 (4)	0.036 (4)	0.035 (4)	0.014 (3)
C25	0.071 (3)	0.091 (4)	0.081 (3)	0.008 (3)	0.006 (3)	0.028 (3)
C26	0.132 (5)	0.079 (4)	0.123 (5)	0.003 (4)	0.030 (4)	0.004 (3)
C27	0.163 (6)	0.138 (6)	0.122 (5)	0.034 (5)	0.076 (5)	0.044 (4)

### Geometric parameters (Å, º)

**Table:**

Li1—C1	2.120 (7)	C13—H13	0.9800
Li1—I1	2.676 (8)	C14—H14A	0.9600
Li1—I1^i^	2.691 (7)	C14—H14B	0.9600
Li1—Li1^i^	3.328 (13)	C14—H14C	0.9600
N1—C1	1.360 (4)	C15—H15A	0.9600
N1—C2	1.386 (5)	C15—H15B	0.9600
N1—C4	1.433 (5)	C15—H15C	0.9600
N2—C1	1.354 (4)	C16—C17	1.389 (6)
N2—C3	1.391 (5)	C16—C21	1.393 (6)
N2—C16	1.440 (5)	C17—C18	1.377 (6)
I1—Li1^i^	2.691 (7)	C17—C22	1.510 (6)
C2—C3	1.328 (5)	C18—C19	1.371 (6)
C2—H2	0.9300	C18—H18	0.9300
C3—H3	0.9300	C19—C20	1.382 (7)
C4—C9	1.382 (5)	C19—H19	0.9300
C4—C5	1.410 (5)	C20—C21	1.378 (6)
C5—C6	1.365 (6)	C20—H20	0.9300
C5—C10	1.507 (6)	C21—C25	1.512 (6)
C6—C7	1.363 (6)	C22—C24	1.509 (6)
C6—H6	0.9300	C22—C23	1.514 (7)
C7—C8	1.371 (7)	C22—H22	0.9800
C7—H7	0.9300	C23—H23A	0.9600
C8—C9	1.384 (6)	C23—H23B	0.9600
C8—H8	0.9300	C23—H23C	0.9600
C9—C13	1.510 (6)	C24—H24A	0.9600
C10—C11	1.497 (8)	C24—H24B	0.9600
C10—C12	1.531 (7)	C24—H24C	0.9600
C10—H10	0.9800	C25—C26	1.525 (7)
C11—H11A	0.9600	C25—C27	1.527 (7)
C11—H11B	0.9600	C25—H25	0.9800
C11—H11C	0.9600	C26—H26A	0.9600
C12—H12A	0.9600	C26—H26B	0.9600
C12—H12B	0.9600	C26—H26C	0.9600
C12—H12C	0.9600	C27—H27A	0.9600
C13—C14	1.509 (7)	C27—H27B	0.9600
C13—C15	1.514 (7)	C27—H27C	0.9600
			
C1—Li1—I1	124.9 (3)	C13—C14—H14B	109.5
C1—Li1—I1^i^	131.6 (4)	H14A—C14—H14B	109.5
I1—Li1—I1^i^	103.4 (2)	C13—C14—H14C	109.5
C1—Li1—Li1^i^	175.8 (5)	H14A—C14—H14C	109.5
I1—Li1—Li1^i^	51.9 (2)	H14B—C14—H14C	109.5
I1^i^—Li1—Li1^i^	51.48 (18)	C13—C15—H15A	109.5
C1—N1—C2	112.3 (3)	C13—C15—H15B	109.5
C1—N1—C4	123.9 (3)	H15A—C15—H15B	109.5
C2—N1—C4	123.6 (3)	C13—C15—H15C	109.5
C1—N2—C3	111.8 (3)	H15A—C15—H15C	109.5
C1—N2—C16	125.3 (3)	H15B—C15—H15C	109.5
C3—N2—C16	122.8 (3)	C17—C16—C21	123.7 (4)
Li1—I1—Li1^i^	76.6 (2)	C17—C16—N2	118.2 (4)
N2—C1—N1	102.8 (3)	C21—C16—N2	118.1 (4)
N2—C1—Li1	134.7 (4)	C18—C17—C16	116.6 (4)
N1—C1—Li1	122.2 (3)	C18—C17—C22	120.5 (4)
C3—C2—N1	106.2 (4)	C16—C17—C22	122.8 (4)
C3—C2—H2	126.9	C19—C18—C17	122.0 (5)
N1—C2—H2	126.9	C19—C18—H18	119.0
C2—C3—N2	106.9 (4)	C17—C18—H18	119.0
C2—C3—H3	126.5	C18—C19—C20	119.4 (5)
N2—C3—H3	126.5	C18—C19—H19	120.3
C9—C4—C5	122.5 (4)	C20—C19—H19	120.3
C9—C4—N1	119.2 (3)	C21—C20—C19	121.8 (5)
C5—C4—N1	118.3 (4)	C21—C20—H20	119.1
C6—C5—C4	117.2 (4)	C19—C20—H20	119.1
C6—C5—C10	121.7 (4)	C20—C21—C16	116.5 (5)
C4—C5—C10	121.1 (4)	C20—C21—C25	120.7 (4)
C7—C6—C5	121.6 (5)	C16—C21—C25	122.8 (4)
C7—C6—H6	119.2	C24—C22—C17	112.6 (4)
C5—C6—H6	119.2	C24—C22—C23	111.7 (5)
C6—C7—C8	120.3 (5)	C17—C22—C23	111.9 (4)
C6—C7—H7	119.8	C24—C22—H22	106.7
C8—C7—H7	119.8	C17—C22—H22	106.7
C7—C8—C9	121.3 (5)	C23—C22—H22	106.7
C7—C8—H8	119.4	C22—C23—H23A	109.5
C9—C8—H8	119.4	C22—C23—H23B	109.5
C4—C9—C8	117.1 (4)	H23A—C23—H23B	109.5
C4—C9—C13	122.8 (4)	C22—C23—H23C	109.5
C8—C9—C13	120.1 (4)	H23A—C23—H23C	109.5
C11—C10—C5	112.0 (5)	H23B—C23—H23C	109.5
C11—C10—C12	108.8 (5)	C22—C24—H24A	109.5
C5—C10—C12	113.4 (5)	C22—C24—H24B	109.5
C11—C10—H10	107.4	H24A—C24—H24B	109.5
C5—C10—H10	107.4	C22—C24—H24C	109.5
C12—C10—H10	107.4	H24A—C24—H24C	109.5
C10—C11—H11A	109.5	H24B—C24—H24C	109.5
C10—C11—H11B	109.5	C21—C25—C26	111.8 (4)
H11A—C11—H11B	109.5	C21—C25—C27	111.0 (5)
C10—C11—H11C	109.5	C26—C25—C27	111.1 (5)
H11A—C11—H11C	109.5	C21—C25—H25	107.6
H11B—C11—H11C	109.5	C26—C25—H25	107.6
C10—C12—H12A	109.5	C27—C25—H25	107.6
C10—C12—H12B	109.5	C25—C26—H26A	109.5
H12A—C12—H12B	109.5	C25—C26—H26B	109.5
C10—C12—H12C	109.5	H26A—C26—H26B	109.5
H12A—C12—H12C	109.5	C25—C26—H26C	109.5
H12B—C12—H12C	109.5	H26A—C26—H26C	109.5
C14—C13—C9	110.7 (4)	H26B—C26—H26C	109.5
C14—C13—C15	109.8 (4)	C25—C27—H27A	109.5
C9—C13—C15	112.4 (4)	C25—C27—H27B	109.5
C14—C13—H13	107.9	H27A—C27—H27B	109.5
C9—C13—H13	107.9	C25—C27—H27C	109.5
C15—C13—H13	107.9	H27A—C27—H27C	109.5
C13—C14—H14A	109.5	H27B—C27—H27C	109.5

Symmetry code: (i) −*x*+1, −*y*+2, −*z*+2.
